# Inter-technique agreement between two-dimensional echocardiography and cardiovascular magnetic resonance imaging in the setting of bicuspid aortic valve disease

**DOI:** 10.1186/1532-429X-16-S1-P129

**Published:** 2014-01-16

**Authors:** Beatriz Miralles Vicedo, Liying Cai, Karima Addetia, Francesco Maffessanti, Kirk T Spencer, Roberto Lang, Victor Mor-Avi, Amit R Patel

**Affiliations:** 1 Cardiology, University of Chicago, Chicago, Illinois, USA

## Background

Bicuspid aortic valve (BAV) is the most common form of congenital heart disease and 30-50% of BAV have an associated aortopathy. Accurate aortic measurements are therefore particularly important in these patients. Two-dimensional echocardiography (2DE) is typically used to follow the aortic size in these patients; however, cardiovascular magnetic resonance (CMR) offers the potential advantage of 3-dimensional datasets that can be used to make more accurate measurements. Inter-technique differences in aortic measurements for BAV patients using these techniques has not been extensively studied. Our aim was to evaluate the measurement variability associated with each technique and their mutual agreement in measuring the aortic root.

## Methods

Retrospective review of patients with BAV who had both CMR and 2DE. Contrast-enhanced magnetic resonance angiography (MRA) or 3D whole-heart imaging (Philips 1.5-T scanner) combined with offline analysis with double oblique planes were used to obtain measurements of the sinus of Valsalva (SOV) on CMR (dimension 1 and 2). On 2DE, the SOV was measured on the parasternal long axis view using the inner-edge to inner-edge technique. Bland-Altman analysis was performed to evaluate the inter-technique agreement between 2DE and CMR. Intraobserver reproducibility was measured as coefficient of variation (CV) and 95% confidence intervals between repeated measurements.

## Results

**Figure 1:**
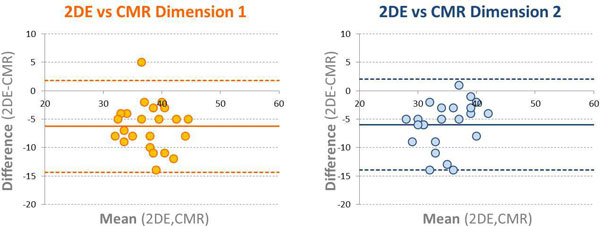


Twenty-three patients (age 40 ± 13 years; range 23-65; 22 men) were studied. The average SOV measurement on 2DE was 35 ± 4 mm, and 41 ± 4 × 41 ± 5 mm on CMR. Correlation between maximal 2DE and CMR diameters was moderate (r = 0.69); Bland-Altman analysis showed significant underestimation by 2DE when compared to CMR dimension 1 (bias = -6.3 mm, LOA = [-14.3-1.8]) and dimension 2 (bias = -6.0 mm, LOA = [-14.0-2.1]) (Figure 1). Both 2DE and CMR were highly reproducible in terms of CV (3.2 and 3.4%, respectively) with narrower confidence interval for CMR (± 2.1 mm) than for 2DE (± 4.8 mm).

## Conclusions

2DE measurements are more variable and significantly underestimate SOV dimensions compared to CMR in patients with BAV. If serial 2DE is being considered to follow aortic root dimensions, CMR should be performed to confirm that the measurement is not significantly underestimating the true maximum diameter.

## Funding

None.

